# Changing the size of a mirror-reflected hand moderates the experience of embodiment but not proprioceptive drift: a repeated measures study on healthy human participants

**DOI:** 10.1007/s00221-017-4930-7

**Published:** 2017-03-18

**Authors:** Priscilla G. Wittkopf, Donna M. Lloyd, Mark I. Johnson

**Affiliations:** 1 Centre for Pain Research, School of Clinical and Applied Sciences, Leeds Beckett University City Campus, Leeds, LS1 3HE UK; 2 School of Psychology, University of Leeds, Lifton Place, Leeds, LS2 9JT UK

**Keywords:** Embodiment, Size distortion, Mirror visual feedback, Multisensory integration

## Abstract

Mirror visual feedback is used for reducing pain and visually distorting the size of the reflection may improve efficacy. The findings of studies investigating size distortion are inconsistent. The influence of the size of the reflected hand on embodiment of the mirror reflection is not known. The aim of this study was to compare the effect of magnifying and minifying mirror reflections of the hand on embodiment measured using an eight-item questionnaire and on proprioceptive drift. During the experiment, participants (*n* = 45) placed their right hand behind a mirror and their left hand in front of a mirror. Participants watched a normal-sized, a magnified and a minified reflection of the left hand while performing synchronised finger movements for 3 min (adaptive phase). Measurements of embodiment were taken before (pre) and after (post) synchronous movements of the fingers of both hands (embodiment adaptive phase). Results revealed larger proprioceptive drift post-adaptive phase (*p* = 0.001). Participants agreed more strongly with questionnaire items associated with location, ownership and agency of the reflection of the hand post-adaptive phase (*p* < 0.001) and when looking at the normal-sized reflection (*p* < 0.001). In conclusion, irrespective of size, watching a reflection of the hand while performing synchronised movements enhances the embodiment of the reflection of the hand. Magnifying and minifying the reflection of the hand has little effect on proprioceptive drift, but it weakens the subjective embodiment experience. Such factors need to be taken into account in future studies using this technique, particularly when assessing mirror visual feedback for pain management.

## Introduction

Mirror visual feedback (MVF) is a technique commonly used in the management of pain and dysfunction, especially in conditions that affect body image, where a body part is perceived as large, swollen, heavy, or stuck in one position, such as complex regional pain syndrome (O’Connell et al. 2013), phantom limb pain (Chan et al. 2007), neuropathy (Moseley 2007) and non-specific back pain (Daffada et al. 2015). MVF is commonly used to manage unilateral limb pain by positioning the non-painful limb in front of a mirror so that it creates a reflection that is aligned with the painful limb positioned behind the mirror. Generally, mirrors that reproduce normal-sized reflections of body parts are used, although mirrors, lenses, binoculars and virtual reality have been used to magnify and minify the size of the painful body part in an attempt to improve the amount of pain relief (Wittkopf and Johnson 2016). To date, there have been no studies that have investigated the effects of magnifying and minifying a mirror reflection of the hand on body perception.

Studies on patients with pain suggest that visual distortion of the size of a body part reduces pain but the direction of the effect may be specific to the individual. Moseley et al. (2008b) found that minifying the appearance of a chronic painful and dysfunctional arm using binoculars alleviated movement-induced pain and reduced swelling in the fingers in a study using ten individuals. Magnifying the appearance of the arm exacerbated movement-induced pain and swelling. Preston and Newport (2011) found that illusory manipulation of the appearance of osteoarthritic hands using real-time video capture techniques was beneficial in 17 out of 20 participants. Interestingly, pain was alleviated by shrinking the appearance of the painful hand in some participants and stretching the appearance of the hand in others.

Studies exposing pain-free, healthy human participants to experimentally induced pain have also shown inconsistent results. In a study using 18 participants, Mancini et al. (2011) found that magnifying a mirror reflection of the hand reduced contact heat pain of the dorsum of the hand whereas minifying the reflected hand increased pain. In contrast, Johnson and Gohil (2016) found no differences in pain associated with immersion of a hand in iced water (cold-pressor pain) when looking at magnified and minified reflections of the hand.

There has been little consideration of the influence of embodiment on outcomes associated with MVF and it is possible that inter-subject variability in embodiment of mirror reflected limbs is contributing to inconsistency in study findings. Embodiment is the subjective experience of the body, including a sense of ownership and agency of body parts (de Vignemont 2011; Longo et al. 2008). Aspects of embodiment have been investigated using the rubber hand illusion (RHI) whereby an individual watches a rubber hand being stroked with a brush whilst their real hand is stroked in synchrony but hidden out of view (Botvinick and Cohen 1998). Within a few minutes the sensation of stroking feels as if it is arising from the rubber hand and the individual experiences a sense that the rubber hand is part of their body (i.e. the rubber hand has been embodied). Embodiment of the rubber hand is accompanied by a sense of ‘loss’ (disembodiment) of the real hand (Lewis and Lloyd 2010) and by physiological responses such as local skin cooling, histamine reactivity, and alterations of neural activity in the brain (Barnsley et al. 2011; Ehrsson et al. 2005, 2004; Lloyd et al. 2006; Moseley et al. 2008a).

As embodiment is a subjective phenomenon it is reliant on self-report and quantified using questionnaires that capture aspects of subjective experience. Investigators have also measured discrepancies in perceived and actual location of body parts during the experimental manipulation, i.e. proprioceptive drift (Tsakiris and Haggard 2005). During the RHI participants report proprioceptive drift of their real hand towards the rubber hand, and the amount of drift is positively correlated with aspects of the subjective embodiment experience (Botvinick and Cohen 1998; Ehrsson et al. 2005, 2004), although sometimes the two measures can be dissociated (Bellan et al. 2015; Lloyd et al. 2013; Rohde et al. 2011). For embodiment to take place the appearance of a body part must fit with an internal body representation model so that a coherent sense of one’s body is maintained (Lewis et al. 2012; Tsakiris et al. 2010). In the RHI, embodiment does not take place if the timing of the tactile stimulus on the real hand and the rubber hand is asynchronous, or if the rubber hand is replaced by an object (i.e. a wooden block) or presented in a non-anatomical position (i.e. rotated 180°, pointing towards the subject) (Botvinick and Cohen 1998; Ehrsson et al. 2005, 2004; Lloyd et al. 2006; Lloyd 2007; Preston 2013; Tsakiris et al. 2010).

The only study investigating the effect of distorting visual appearance of the size of the hand on aspects of embodiment was conducted by Pavani and Zampini (2007). During the experiment, a video monitor was placed horizontally on a table off-centre to the left of the participant’s mid-sagittal plane, and a real-time video showed their left hand being stroked by a brush. The size of the video image was manipulated by magnifying and minifying the participant’s left hand, which was placed 25 cm from the monitor and hidden from view. The authors found that participants reported a proprioceptive drift of their real hand (out of view) towards the video monitor only when observing a normal and a magnified image of their hand, but not when looking at a minified image of their hand.

There have been few studies that have investigated embodiment during MVF and those that exist have used mirrors that create a normal-sized reflection of the body part. Holmes et al. (2004) and Holmes and Spence (2005) found that looking at the reflection of the hand during MVF had an effect on perceived location of the hand hidden behind the mirror. Participants reported proprioceptive drift of their hand hidden behind the mirror towards their body and the mirror when looking at the reflection of the hand but not when looking at a mirror covered with cardboard. The magnitude of the proprioceptive drift was increased by active synchronised movements of both hands and by increasing duration of exposure to MVF. Recently, Medina et al. (2015) found that movement of a hand behind the mirror whilst watching a reflection of a hand moving in synchrony in front of the mirror increased the intensity of embodiment as measured using a questionnaire. Synchronous movements of hands also increased the magnitude of proprioceptive drift of the hand behind the mirror towards the reflection of the hand. Thus, visuo-motor experience and time of exposure appear to influence embodiment of reflections of body parts during MVF using normal-sized reflections of body parts.

To our knowledge, there have been no studies investigating the influence of magnifying and minifying the reflection of a body part on embodiment. The aim of our study was, therefore, to compare the effect of magnifying and minifying mirror reflections of the hand on embodiment measured using an eight-item embodiment questionnaire and on proprioceptive drift. We hypothesised that there would be differences in both outcome measures between a normal-sized reflection of the hand compared with magnified and minified reflections of the hand but the direction of these differences could not be anticipated because of the inconsistences in previous research.

## Methods

### Study design

A within-subject repeated-measures design was used to compare measures of embodiment of a normal-sized reflection, magnified reflection, and minified reflection of the hand. Each participant took part in one experiment that measured embodiment of their reflected hand under three conditions, with the order of presentation of conditions randomised:

- Normal-sized reflection of the left hand using a 46-cm diameter flat mirror
- Magnified reflection of the left hand using a 46-cm diameter concave mirror (1.3 magnification)
- Minified reflection of the left hand using a 46-cm diameter convex mirror (0.7 magnification).

Embodiment of the mirror reflection of the hand was facilitated using a sequence of synchronised movements of the real hands behind and in front of the mirror, which we termed the adaptive phase. Ethical approval was received from the Research Ethics Committee of Leeds Beckett University.

### Participants

A convenience sample of 45 unpaid volunteers aged 18 years or above was sought via announcements in lectures in our university. Volunteers were requested not to take part in the study if they did not consider themselves healthy, had a long-term illness, were currently seeking medical care, were experiencing pain or sensory disturbances, taking any medication, were known to be pregnant, had a dermatological condition or were unable to see clearly at a distance of up to 1 m. There was no restriction on gender, ethnicity nor body mass index although this was recorded. Volunteers expressing interest received a participant information pack and were given 48 h to consider participation before a formal invitation to attend a study visit was made. During the study, visit volunteers were formally screened for eligibility and then provided written consent. Participants were reminded that they could withdraw consent at any time and without reason.

### Experimental procedure

Each participant attended our research laboratory for one experimental visit lasting no longer than 2 h. Each experiment was conducted by the principal investigator (PW: 27 years old, female, physiotherapist, Brazilian national) who is fluent in English. All instructions were read verbatim from a crib sheet to ensure that all participants received standardised information.

**Fig. 1:**
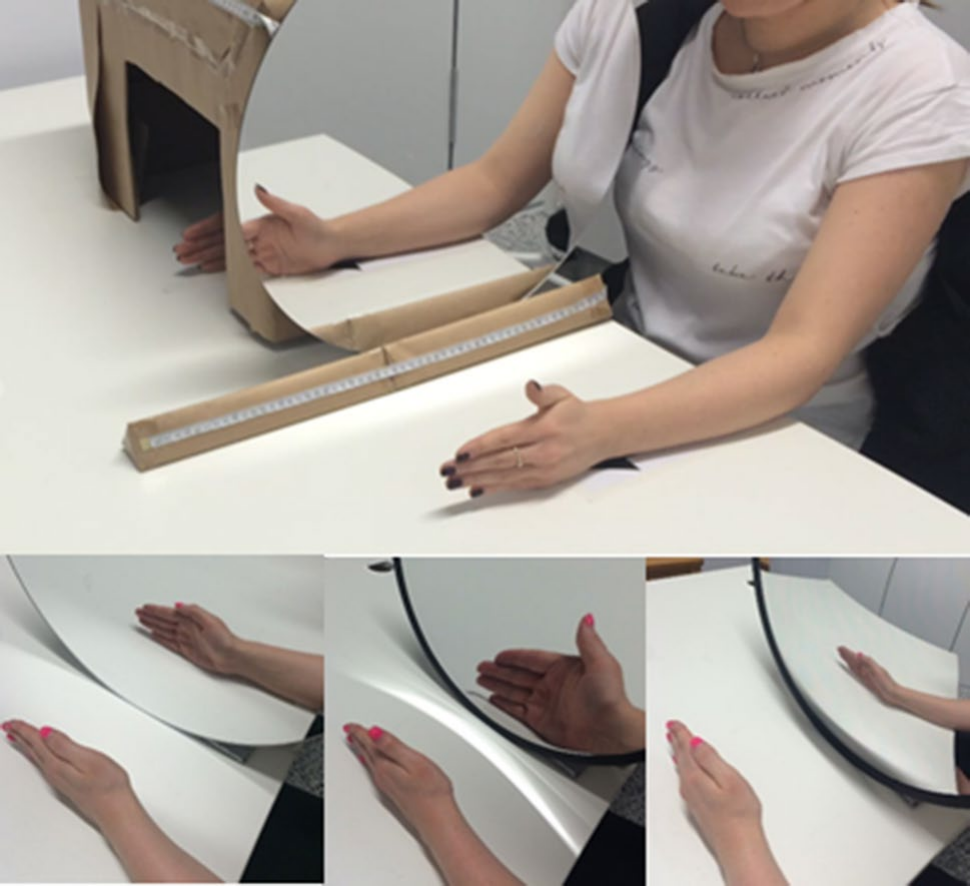
Experimental set up (*top*). Three experimental conditions, normal-sized reflection, magnified reflection and minified reflection in order (*bottom*)

Measurements of the participant’s height, weight and left hand size (from wrist perpendicular to the scaphoid to the tip of the middle finger) were taken. Participants were seated with both arms resting on a desk, flexed at the elbows with the right hand placed in a cardboard box at a distance of 25 cm behind a mirror attached to the outer left hand wall of the box (Fig. 1). A black cloth was draped across the participant’s right arm onto the shoulder and the left hand placed 25 cm in front of the mirror. Initially, a cardboard covered the reflective surface of the mirror. Both hands were in a neutral position and the fingers straight in line with the palms.

**Fig. 2:**
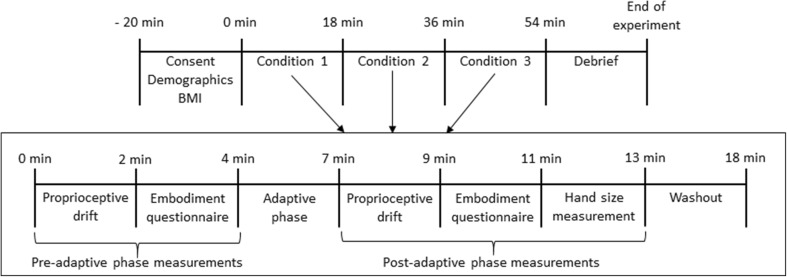
Time-course of the overall experiment and of one condition

Measurements of embodiment were taken for three mirror conditions (normal-sized, magnified, minified) during three identical measurement cycles, with the order of presentation of the conditions randomised. Each cycle started with ‘pre-adaptive phase measurements’ (Fig. 2). These consisted of measurement of proprioceptive drift with the mirror covered by the cardboard. Then the mirror was uncovered and participants were instructed to look at the reflection of their left hand and provide a verbal responses to questions read from the embodiment questionnaire by the investigator. This was followed by an ‘adaptive phase’ that attempted to facilitate embodiment of the reflected hand. Participants were asked to look at the reflection of their left hand for 30 s, followed by clenching and unclenching of both fists in synchrony with a metronome (60 beats per minute) for 60 s. This was followed by keeping both hands still for 30 s followed by 60 s of touching each fingertip with the thumb in synchrony with a metronome (60 beats per minute). Immediately after the ‘adaptive phase’ participants were asked to look at the reflection of their left hand and ‘post-adaptive phase’ measurements of proprioceptive drift and answers to the embodiment questionnaire were taken. The participant’s estimation of the size of the reflection of their left hand was measured using a ruler placed parallel to the mirror. Participants were asked to state the numbers on the ruler that were in line with the reflection of the tip of their middle finger and the mark on their wrist. Participants then rested for 5 min before commencement of the next measurement cycle.

### Outcome measures

#### Proprioceptive drift

Participants were asked to say “stop” when proprioceptive awareness of their middle finger of the hand hidden behind the mirror coincided with the position of a pen that was being moved by the investigator along the top of the cardboard box in the left–right axis. The distance (cm) between the perceived location and actual location of the hidden hand was measured using a ruler and recorded as proprioceptive drift.

#### Embodiment questionnaire

The embodiment questionnaire was based on previous studies (Lewis and Lloyd 2010; Longo et al. 2008; Medina et al. 2015). Participants were asked to rate each of the following statements by stating a whole number from 0 = strongly disagree to 10 = strongly agree:

- It feels as if my right hand is in the same location as the reflection of the hand (associated with Location of body part)
- It feels like I am looking directly at my right hand rather than at a reflection of the hand (associated with Ownership of the reflection)
- It feels as if the reflection of the hand is my real hand (associated with Ownership of the reflection)
- It feels as if the reflection of the hand is part of my body (associated with Ownership of the reflection)
- It feels as if I could move the reflection of the hand without having to move my left hand (associated with Agency of the reflection)
- It feels as if I move my right hand the reflection of the hand will move too (associated with Agency of the reflection)
- It feels like I cannot tell where my right hand is (associated with Deafference)
- My right hand feels unusual (associated with Deafference).

### Data analysis

A 3 × 2 repeated measures factorial analysis of variance (ANOVA) was conducted on proprioceptive drift and embodiment questionnaire data. Within-subject factors were condition (three levels: normal-sized, magnified, minified) and time (two levels: pre-adaptive phase and post-adaptive phase). Between-subject factors were order (six levels: normal, minified, magnified; minified, magnified, normal; magnified, normal, minified; magnified, minified, normal; normal, magnified, minified; minified, normal, magnified). A Greenhouse–Geisser correction was used if Mauchly’s test showed that sphericity could not be assumed. Adjustments were made for multiple comparisons using the Bonferroni correction. The level of statistical significance was set at *p* ≤ 0.05 and power ≥ 0.80. When a significant interaction was detected and power was greater than 0.80, simple effect analyses were conducted to determine the direction of the interaction. Analyses were conducted with SPSS version 22.0 and G*Power 3.1.

## Results

### Characteristics of study sample

Forty-five (female *n* = 30) right-handed volunteers expressed interest in the study and all started and completed the experiment (mean ± SD: age = 22.69 ± 6.83 years, weight = 67.03 ± 12.53 Kg; height = 1.70 ± 0.08 m).

### Estimation of the size of reflection of the left hand

The mean + SD size of the real hand was 18.84 ± 1.14 cm. The main effect of condition was statistically significant [*F*(1.78,78.58) = 49.58, *p* < 0.001, $\eta _{p}^{{\text{2}}}$ = 0.530, power = 1.0]. Pairwise comparisons found that participants estimated that both the normal-sized reflection (16.25 ± 2.17 cm, *p* < 0.001) and the minified reflection (12.55 ± 4.27 cm, *p* < 0.001) of the hand to be smaller than the size of the real hand. There was no difference between estimates for the size of the magnified reflection (20.24 ± 4.80 cm, *p* = 0.344) compared with the real hand. There was a significant difference between the three conditions on estimation of the size of the reflection of the hand (i.e. normal-sized vs. magnified *p* < 0.001; normal-sized vs. minified *p* < 0.001; and magnified vs. minified *p* < 0.001).

### Proprioceptive drift

**Fig. 3:**
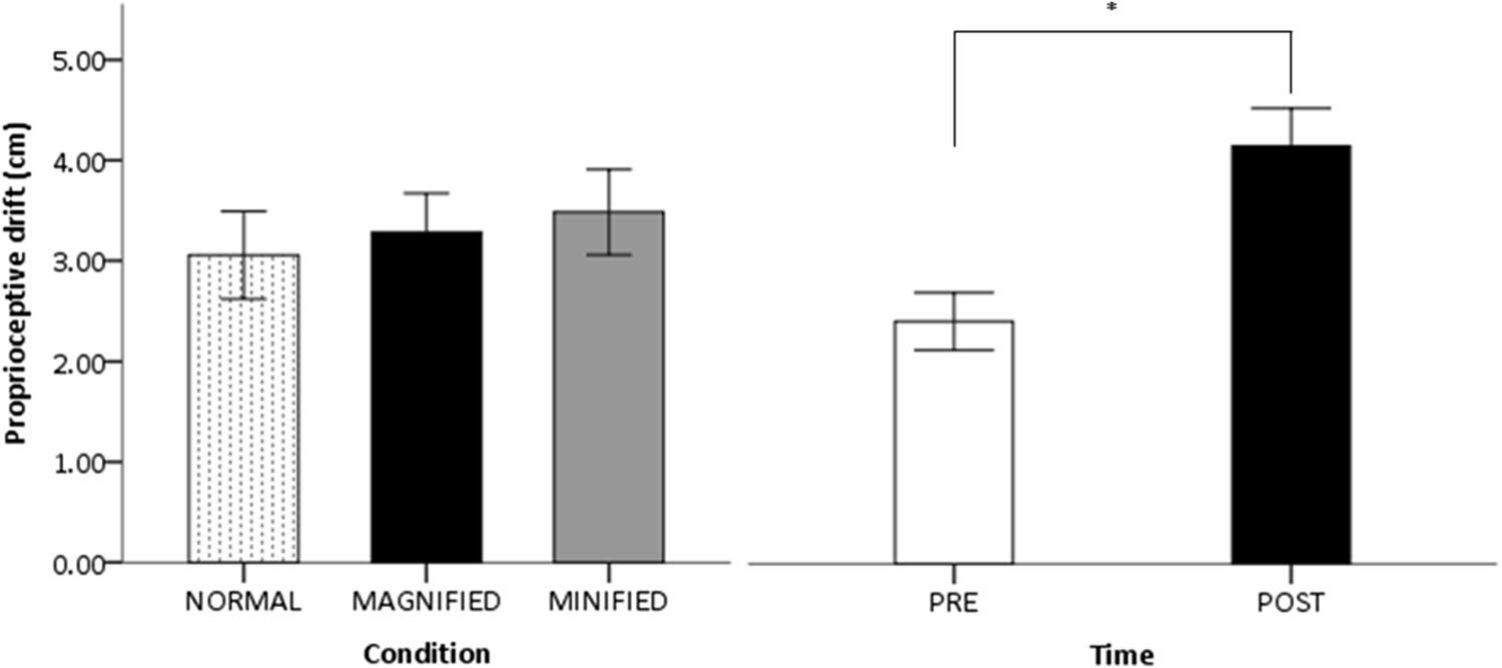
Mean proprioceptive drift. *Error bars* indicate standard errors of the mean. Results separated by condition (*left*) and time (*right*). *Asterisk* shows significant difference at *p* = 0.001 with pairwise comparisons

The main effect of time was statistically significant [*F*(1,44) = 16.369, *p* = 0.001, $\eta _{p}^{{\text{2}}}$ = 0.332, power = 0.996, Fig. 3], indicating that participants experienced larger proprioceptive drift in the post-adaptive phase compared with the pre-adaptive phase. This suggests that synchronised movements of fingers increased proprioceptive drift. There were no significant interactions (all *F*s < 2.1, n.s.). The main effect of condition was not statistically significant [*F*(2,78) = 0.376, *p* = 0.688, $\eta _{p}^{{\text{2}}}$ = 0.010, power = 0.10, Fig. 3], indicating that the magnitude of proprioceptive drift of the hidden hand experienced by the participants did not differ between the three sizes of reflected hand.

### Embodiment questionnaire

**Fig. 4:**
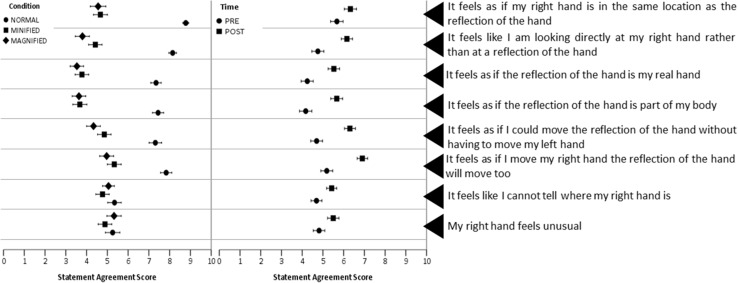
Mean embodiment questionnaire scores, *error bars* indicate standard errors of scores on a numerical rating scale where *0* strongly disagree and *10* strongly agree separated by condition (*left*) and time (*right*)

Visual inspection of summary data suggested that participants agreed more strongly with the statements after the adaptive phase (Fig. 4) and when looking at the normal-sized reflection compared with the magnified and minified reflections (Fig. 4). This was confirmed by the ANOVA that found that the main effect of condition and of time was statistically significant (Table 1).

**Table 1:** Analysis of variance for the items in the embodiment questionnaire (only the statistically significant main effects and interactions are presented)

Questionnaire statement	ANOVA factors	*df*	*F*	*p*	$\eta _{p}^{{\text{2}}}$	Power
It feels as if my right hand is in the same location as the reflection of the hand	Condition	1.63, 63.75	47.94	0.001	0.551	1.00
	Time	1,39	27.16	0.001	0.411	0.99
	Condition × order	8.17,63.75	3.59	0.002	0.315	0.85
It feels like I am looking directly at my right hand rather than at a reflection of the hand	Condition	2,78	67.19	0.001	0.633	1.00
	Time	1,39	30.19	0.001	0.436	0.99
	Condition × order	10,78	2.07	0.037	0.209	0.82
It feels as if the reflection of the hand is my real hand	Condition	2,78	47.97	0.001	0.552	0.99
	Time	1,39	17.85	0.001	0.314	0.99
It feels as if the reflection of the hand is part of my body	Condition	2,78	50.09	0.001	0.562	1.00
	Time	1,39	29.19	0.001	0.428	0.99
	Condition × order	10,78	2.18	0.027	0.219	0.81
It feels as if I could move the reflection of the hand without having to move my left hand	Condition	2,78	28.15	0.001	0.429	0.99
	Time	1,39	26.14	0.001	0.401	0.99
It feels as if I move my right hand the reflection of the hand will move too	Condition	2,78	28.96	0.001	0.426	0.99
	Time	1,39	34.56	0.001	0.470	0.99
	Condition × time × order	10,78	2.30	0.020	0.228	0.81
It feels like I cannot tell where my right hand is	Time	1,39	7.21	0.01	0.156	0.85
My right hand feels unusual	Time	1,39	6.53	0.01	0.144	0.80

*df* the degrees of freedom, *F* result of the ANOVA test, *p* is the significance level, $\eta _{p}^{{\text{2}}}$ is the effect size

In all instances where the main effect of time was statistically significant participants agreed more strongly with the statement when it was presented during the post-adaptive phase compared with the pre-adaptive phase (*p* < 0.001). In all instances where the main effect of condition was statistically significant participants agreed more strongly with the statement when looking at the normal-sized reflection compared with the magnified (*p* < 0.001) or minified reflections (*p* < 0.001).

**Fig. 5:**
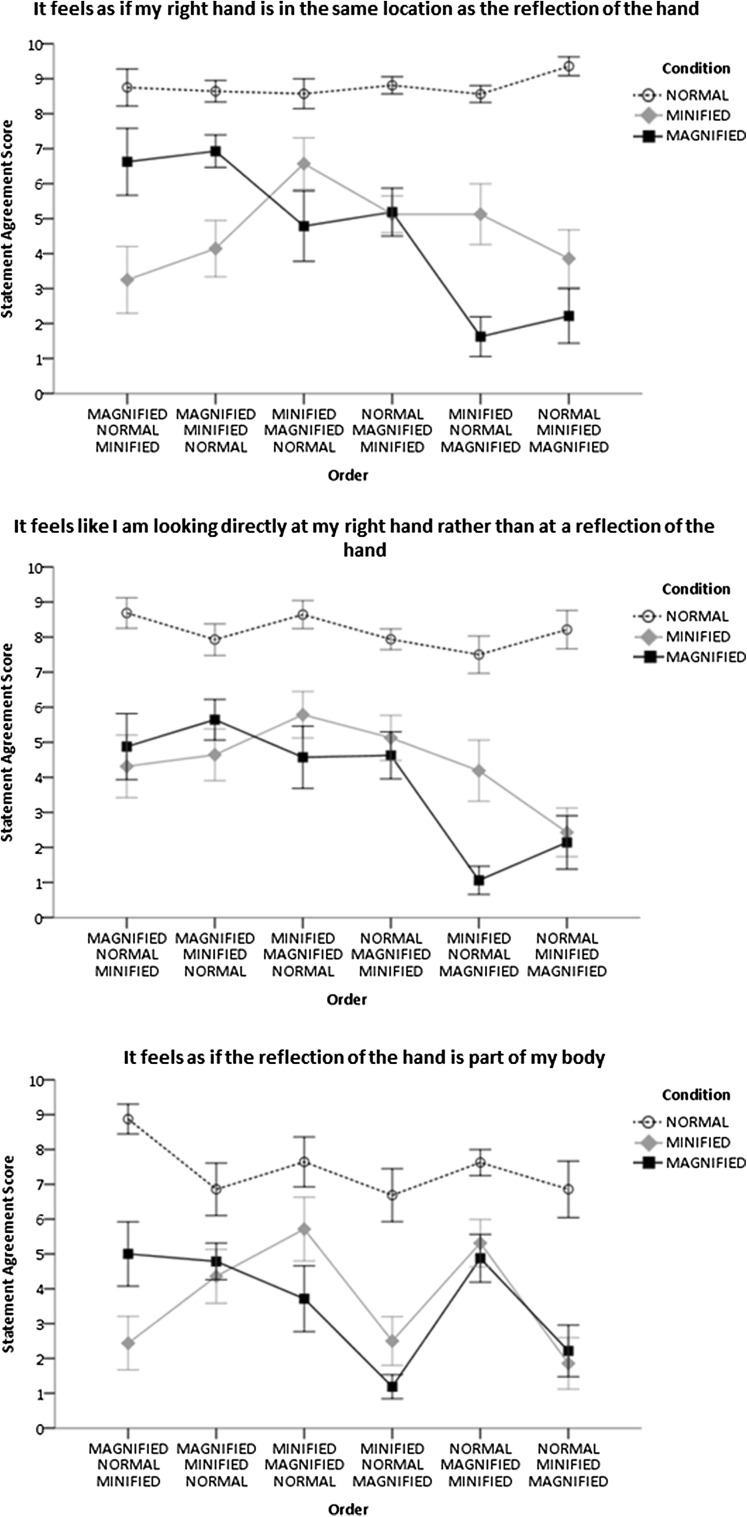
The interaction between condition and order. Mean and standard errors of scores on a Likert scale where *0* strongly disagree and *10* strongly agree

There was a statistically significant interaction between condition and order for the statements ‘It feels as if my right hand is in the same location as the reflection of the hand’ and ‘It feels like I am looking directly at my right hand rather than at a reflection of the hand’. This indicated that the strength of agreement at that moment in time was greater if the magnified reflection was presented before the normal-sized or minified reflections (Fig. 5). The statistically significant interaction between condition and order for the statement ‘It feels as if the reflection of the hand is part of my body’ indicated equally strong agreement for the statement when looking at the normal-sized, minified and magnified reflections of the hand if the order of presentation of conditions was magnified, minified and normal-sized condition (Fig. 5). The statistically significant interaction between condition, order and time for the statement ‘It feels as if I move my right hand the reflection of the hand will move too’ indicated equally strong agreement of the statement when looking at the normal-sized, minified and magnified reflections of the hand in the post-adaptive phase.

## Discussion

### Summary of findings

Our study provides evidence that proprioceptive drift towards the midline of the body occurs in a hand hidden behind a mirror after watching a mirror reflection of the other hand performing finger movements synchronised with movements of the hidden hand. This visuo-motor stimulus increased the perceptual experience that the hidden hand was in the same location as the reflection of the hand and that the reflection of the hand was the participant’s real hand (ownership) and could be moved without having to move the hand hidden behind the mirror (agency). These findings suggest that, irrespective of the size of the reflection of the hand, the experience of embodying a reflection of the hand was enhanced by watching the reflection whilst performing synchronised movements of both hands over a 3-min time period.

Our study provided tentative evidence that magnifying and minifying the reflection of the hand may weaken embodiment when compared with a normal-sized reflection of the hand. When viewing magnified and minified reflections participants in our study agreed less strongly that their hidden hand was in the same location as the reflection of the hand and that the reflection of the hand was the participant’s real hand and could be moved without having to move the hand hidden behind the mirror. Magnifying or minifying the size of the reflection of the hand did not influence estimates of the magnitude of proprioceptive drift, suggesting dissociation between proprioceptive drift and the sense of ownership and agency of the reflection of the hand (see also Lloyd et al. 2013). There was an underestimation of perceived hand size when participants were looking at the normal-sized reflection of the hand. This could be related to the underestimation of the finger length in proprioceptive and visual matching measures as previously reported by Longo and Haggard (2010) and Longo and Haggard (2012).

Responses to some statements about embodiment were influenced by a combination of condition and the order of presentation of the conditions. When looking at the magnified reflection of the hand the strength of agreement at that moment in time for some statements was greater if the magnified reflection was presented before the normal-sized or minified reflections (i.e. “It feels as if my right hand is in the same location as the reflection of the hand” and “It feels like I am looking directly at my right hand rather than at a reflection of the hand”). A possible interpretation of this finding is that the first reflection acted as a reference on which subsequent participants made judgements about embodiment and the priming influence of the magnified reflection, when presented first, was stronger than that of normal-sized and minified reflections. In addition, a significant condition × time × order interaction showed that participants agreed equally strongly with the statement “It feels as if I move my right hand the reflection of the hand will move too” when looking at the normal-sized, minified and magnified reflections of the hand and post-adaptive phase. This may indicate that the synchronised movements of the fingers equally enhanced the experience of agency of the reflection of the hand when looking at the normal-sized, magnified and minified reflection of the hand.

### Previous studies of visual distortion and embodiment

To date, only one study has investigated the effect of distorting the size of a body part on embodiment. Pavani and Zampini (2007) used real-time video images to manipulate the size of a real hand so that it appeared reduced, enlarged, or the same size (veridical) when compared with their real hand. It was found that the felt location of the real hand was biased towards the location of veridical and enlarged, but not reduced video images. They suggested that multisensory modulation of the body schema tended to acknowledge enlarged but not reduced images of body parts within our body representation. In our study, there was a tendency for proprioceptive drift of the real hand towards the midline of the body (i.e. the location of a mirror) but there were no differences in the magnitude of this drift between magnified, minified and normal-sized reflections of the hand. The use of different techniques to distort hand size and the use of different stimuli to facilitate embodiment during the adaptation phase may explain in part the difference in findings with Pavani and Zampini (2007). Pavani and Zampini (2007) used visuo-tactile stimuli by brushing participant’s finger whilst they observed synchronous brush strokes on the real-time video image. We elected to use synchronous sequential finger movements to facilitate embodiment because this technique is used in the rehabilitation of injured and painful hands. Furthermore, creating an illusion of brushing on a reflection of the hand requires simultaneous brushing of the hand in front of the mirror, which would create a tactile input which may distract the participant. It is possible to create an illusion of stroking the surface of a hand reflected in a mirror whilst making stroking motions of a brush held above the hand in front of the mirror. This requires careful alignment of the position of the participant’s view of the reflection and the illusion can be broken with even small movements of the participant’s head.

### Facilitating embodiment

The findings of studies that have used visuo-motor stimuli to facilitate embodiment of a virtual hand or mirror reflection of the hand show that the magnitude of proprioceptive drift is larger when there is greater spatial temporal congruence between the real and the viewed hand and longer periods of exposure (Asai 2016; Holmes et al. 2006; Holmes and Spence 2005; Romano et al. 2015; Sanchez-Vives et al. 2010; Tsakiris et al. 2006). For example, Asai (2016) found that spatial congruence between performed and observed movements were essential for proprioceptive drift. Participants observed a real-time video of their hand whilst they performed active movements. The spatial orientation of the hand was manipulated to create an incongruent condition where the viewed hand was flipped, so that the palm was facing up and the palm of the real hand facing down. The perception of the location of the real hand was closer to the monitor during congruent but not incongruent conditions. In addition, they found proprioceptive drift of a real hand towards the monitor when LEDs were attached to the fingers and thumb so that participants were able to observe points of light rather than hand shape during movements, suggesting that the body shape may not be necessary for proprioceptive drift to occur. Interestingly, participants did not report feelings of ownership or agency suggesting a plausible image is necessary for the subjective experience of embodiment of the viewed body part. In our study, the position of the participants’ hands and synchronised movements were standardised for all conditions during the adaptive phase reducing the effect of spatial orientation on outcome. Moreover, there were no tasks involving reaching points, or object manipulation, which are known to be influenced by the perceived size of the hand. Thus, recalibration of the representation of the hand was not a necessary requirement when judging the location of the hand (i.e. proprioceptive drift) (Bernardi et al. 2013; de Vignemont 2011; Linkenauger et al. 2013).

### Dissociation of proprioceptive drift and ownership and agency of the embodiment experience

Our findings, that magnifying and minifying the mirror reflection of the hand had minimal effect on proprioceptive drift but weakened feelings of ownership, agency, and location of the reflection of the hand, add to a growing body of evidence of dissociation between proprioceptive drift and ownership and agency of the embodiment experience. Our study is the first to observe this phenomenon in different sized reflections of body parts, although dissociation has been reported previously in studies using the RHI, normal-sized reflection of body parts, real-time video of body parts and robotic hands (Asai 2016; Bertamini and O’Sullivan 2014; Holmes et al. 2006; Kammers et al. 2009; Lewis et al. 2012; Lloyd et al. 2013; Rohde et al. 2011; Romano et al. 2015; Shimada and; Hiraki 2009; Tsakiris and Haggard 2005). Rohde et al. (2011) found that the strength of the embodiment experience only increased when participants watched the rubber hand being stroked in synchrony with their real hand, whereas proprioceptive drift occurred during both synchronous and asynchronous stroking. Sometimes proprioceptive drift occurred without the feeling of ownership of the rubber hand. Romano et al. (2015), using a detached myoelectric-controlled robotic hand, found that participants reported a proprioceptive drift of their real hand towards the robotic hand when both were moving in synchrony, but no feelings of ownership were present. It was suggested that this dissociation could arise because the robotic hand was aesthetically different from a real hand. Holmes et al. (2006) suggested that areas in the brain responsible for integrating visual and proprioceptive information only have access to very basic visual information concerning body parts. This visual information may specify only the approximate shape, size, and position of the hand, yet may still be sufficient to begin the process of recalibrating the felt location of the hand, but is not enough to produce ownership.

It has been suggested that while proprioceptive drift would be related to a modification of the body schema, feelings of ownership would be related to the body image (de Vignemont 2010, 2011; Romano et al. 2015). The dissociation between proprioceptive drift and the subjective experience of embodiment is consistent with the dissociation of body schema, a non-conscious performance of the body in the environment, and body image, i.e. conscious awareness of one’s own body (de Vignemont 2010, 2011; Gallagher 1986, 2005; Kammers et al. 2009). The concept of body image involves at least three aspects: perceptual, how I perceive my body; cognitive, what I know about my body; emotional, what I feel about my body (Gallagher 1986, 2005). Moreover, body image is considered a stable representation of long-term bodily properties such as the size of the various body parts, meaning that modifications to this representation are not part of a dynamic online update process (de Vignemont 2010, 2011). In the present study, participants had stronger feelings of ownership and agency when looking at the normal-sized reflection of the hand in comparison with the magnified and minified conditions. This indicates that the short period of exposure to the conditions did not change participants’ feelings of ownership and agency towards the hand and that the subjective experience of the embodiment is more related to body image. An integration of the concepts of body image and body schema has been proposed by Moseley et al. (2012). They propose that the body matrix is the neuronal circuitry of the cortex that processes information from areas of the brain that code for visual, tactile, and proprioceptive input, and underpins the multisensory representation of the body and the space around it. Distorting the size of the reflection may disrupt the sense of unity of the body, thus reducing a sense of ownership of magnified and minified reflections of the hand.

### Limitations of the study

#### Depth perception

It is possible that depth perception is confounding the measurements of embodiment in studies using mirror reflections and other magnifying and minifying techniques (e.g. binoculars, virtual reality). Concave and convex mirrors and lenses create images of body parts that look closer or further away from the eyes of the participant. The location of the image depends on the location of the real body part and the curvature, focal point and length of the mirror or lens. The size of a virtual body part and the size of the image during real-time video capture of a body part are also manipulated by changing the distance between the participant’s eyes and the image (i.e. zooming in or zooming out). That means that, in a three dimensional view, changes in the size of the viewed body part is proportional to changes in the depth perception of the image. A minified hand will be seen further away from the participant’s eyes, whereas a magnified hand will be seen closer to the participant’s eyes. It is known that during the RHI it is more difficult to embody the rubber hand when it is placed further away from the participant’s midline (Aimola Davies et al. 2013; Lloyd 2007). The effect of changing depth perception of the viewed body part on the embodiment experience has not been investigated yet and could be a confounding factor when measuring the embodiment experience of magnified and minified images of body parts. It is important to address this aspect in future studies to isolate the effects of the size of manipulation of the body part and the depth perception of the viewed body part.

#### Accuracy of subjective reports

The phenomenon of embodying a rubber hand and the outcome measures used to quantify this experience have been adapted to be used during MVF. However, the translation of the phenomenon of embodying a rubber hand to embodying a reflection of the hand can be problematic. For example, the statement ‘It feels as if the reflection of the hand is my real hand’ can be confusing as it relates to the reflection of the participant’s own hand. This is a big difference from the RHI, where participants know the rubber hand is not their own hand. Although the questionnaire captures more aspects of the subjective experience of embodiment, it has more cognitive bias than measurement of proprioceptive drift (Asai 2016). Therefore, the accuracy of the subjective reports should be considered carefully when interpreting the findings of studies investigating the embodiment of a reflection of a body part. A limitation of our study (and many other studies using this technique) was that the investigator was not blinded to the conditions when measuring proprioceptive drift and this has the potential to introduce experimenter-expectancy bias related to the investigator’s cognitive bias subconsciously influencing participants and measurements.

An interesting finding from our study was that the priming influence of the magnified reflection, when presented first, was stronger than that of normal-sized and minified reflections. This indicates that the order in which the conditions are presented may affect the embodiment experience of the reflection of the hand and should be taken into account when designing future studies.

#### Implications for MVF

Studies investigating the effect of visually distorting the size of a body part have given small consideration to the embodiment of the viewed body part. Studies have used questionnaires to measure the subjective experience of embodiment, and no study has used proprioceptive drift as a measure of embodiment of the viewed body part. Mancini et al. (2011) and Johnson and Gohil (2016) used mirrors to magnify and minify the reflection of the participants hand while experimentally inducing pain on the hand hidden behind the mirror. The authors used two questions associated with ownership of the reflected hand and found that participants agreed equally strongly to the questions when looking at the normal-sized, magnified and minified reflections of the hand. Recently, Romano et al. (2016) exposed 21 healthy volunteers to blunt pressure pain while looking at a virtual leg from a first person perspective and compared this to a magnified and minified view of the virtual leg. The authors used questions associated with ownership, agency and location of the virtual leg and found a significant but underpowered main effect of size for the question associated with location. The lack of a standardised outcome measure to quantify the embodiment of the viewed body part leads to inconsistent conclusions. Therefore, it is not clear if participants are embodying the magnified and minified images of the body part and if differences in pain perception are related to the manipulation of the size of the viewed body part. The results of our study suggest that it is important that future studies use more complete outcome measures to control for the subjective experience of embodiment of a magnified and minified image of a body part.

## Conclusion

In conclusion, our study provides evidence that the experience of embodiment is enhanced after watching a mirror reflection of a hand performing finger movements synchronised with movements of the other hand hidden behind the mirror. Magnifying and minifying the reflection of the hand has little effect of proprioceptive drift, but weakens the subjective embodiment experience, measured with questionnaire statements. Furthermore, we found that the order in which the conditions are presented can affect the embodiment experience and such factors need to be taken into account in future studies using this technique, particularly when assessing MVF as a clinical intervention for the management of pain and pain-related dysfunction.
